# Supplementary figures and images for: Down-regulated NEDD4L facilitates tumor progression through activating Notch signaling in lung adenocarcinoma
Source: PeerJ. 2022 May 24;10:e13402. doi: 10.7717/peerj.13402 (PMC9138047; doi:10.7717/peerj.13402)

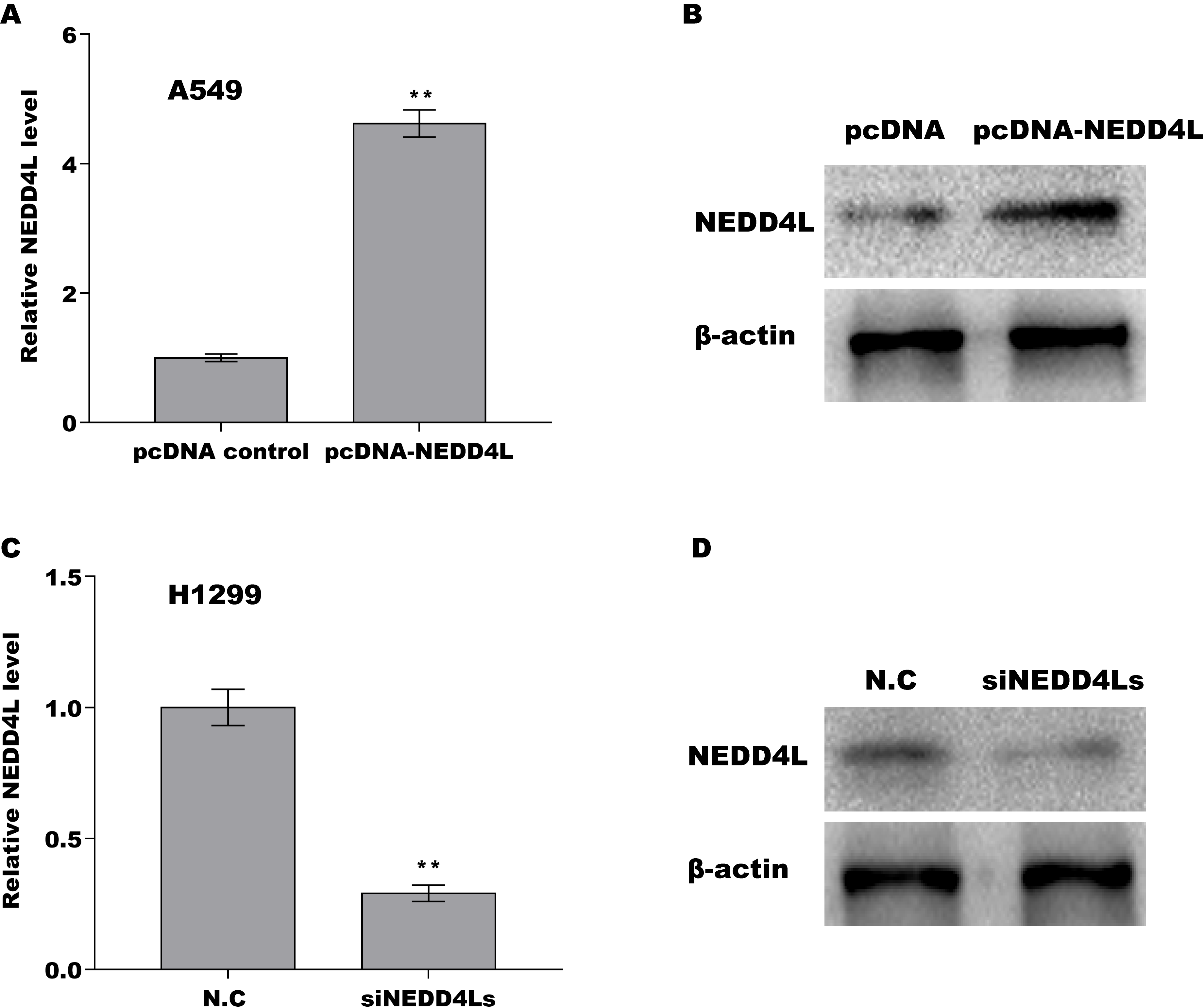

Supplement: Supplemental Information 3 — Recombinant plasmids (pcDNA-NEDD4L) were transfected into A549 cells, and then the mRNA (A) and protein (B) levels of NEDD4L were assessed using qRT-PCR and western blot analysis. siRNAs against NEDD4L (siNEDD4Ls) were transfected into H1299 cells, and then the mRNA (C) and protein (D) levels of NEDD4L were assessed using qRT-PCR and western blot analysis. [file peerj-10-13402-s003.tif]

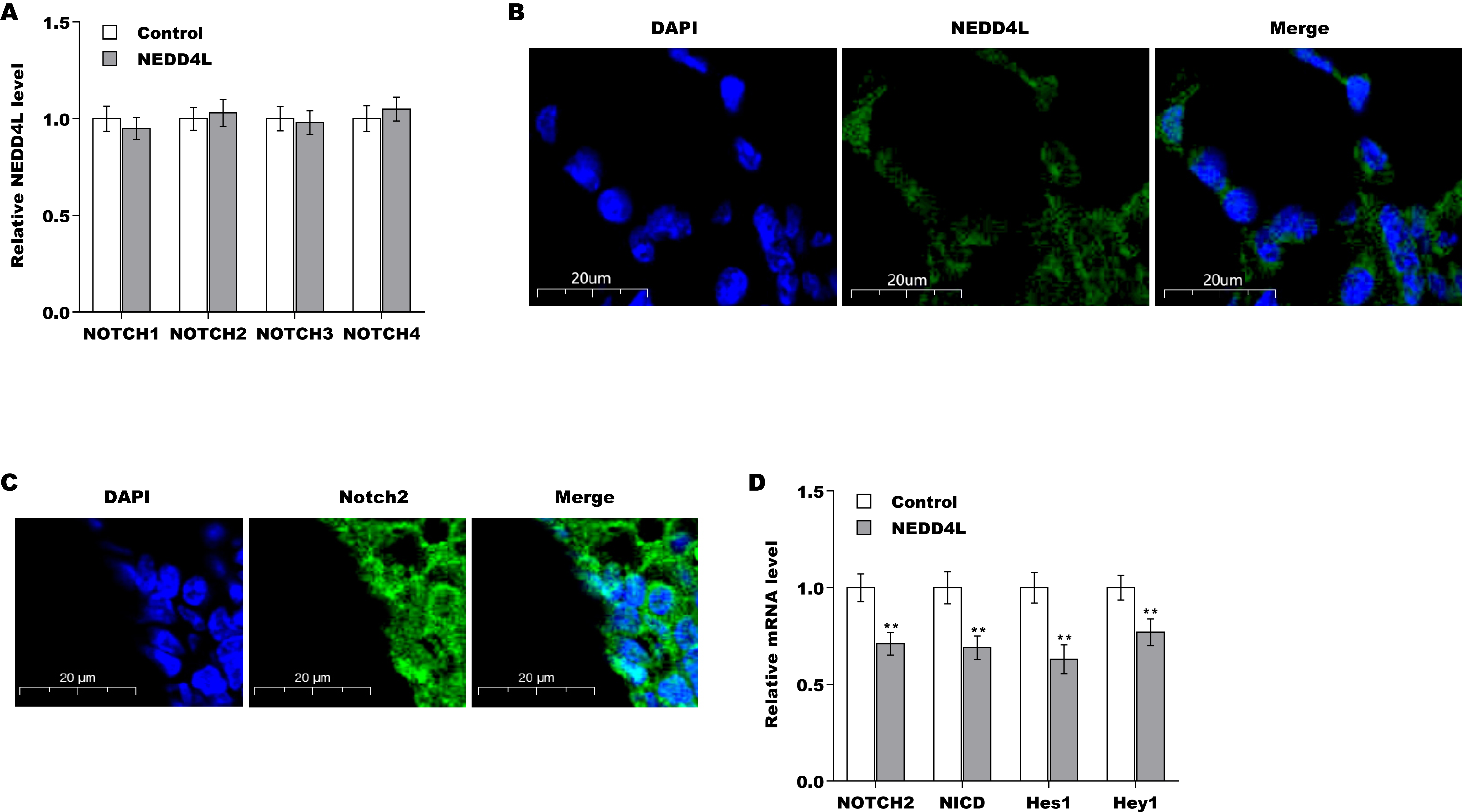

Supplement: Supplemental Information 4 — (A) qRT-PCR was carried out to assess NOTCH1-4 mRNA level in A549 cells after NEDD4L overexpression. Immunofluorescence analysis was carried out to assess the cellular localization of NEDD4L (B) and NOTCH2 (C) in A549 cells. (D) The mRNA levels of NOTCH2, NICH, Hes1, and Hey1 was assessed using qRT-PCR analysis in A549 cells after NEDD4L overexpression. **p < 0.01. [file peerj-10-13402-s004.tif]

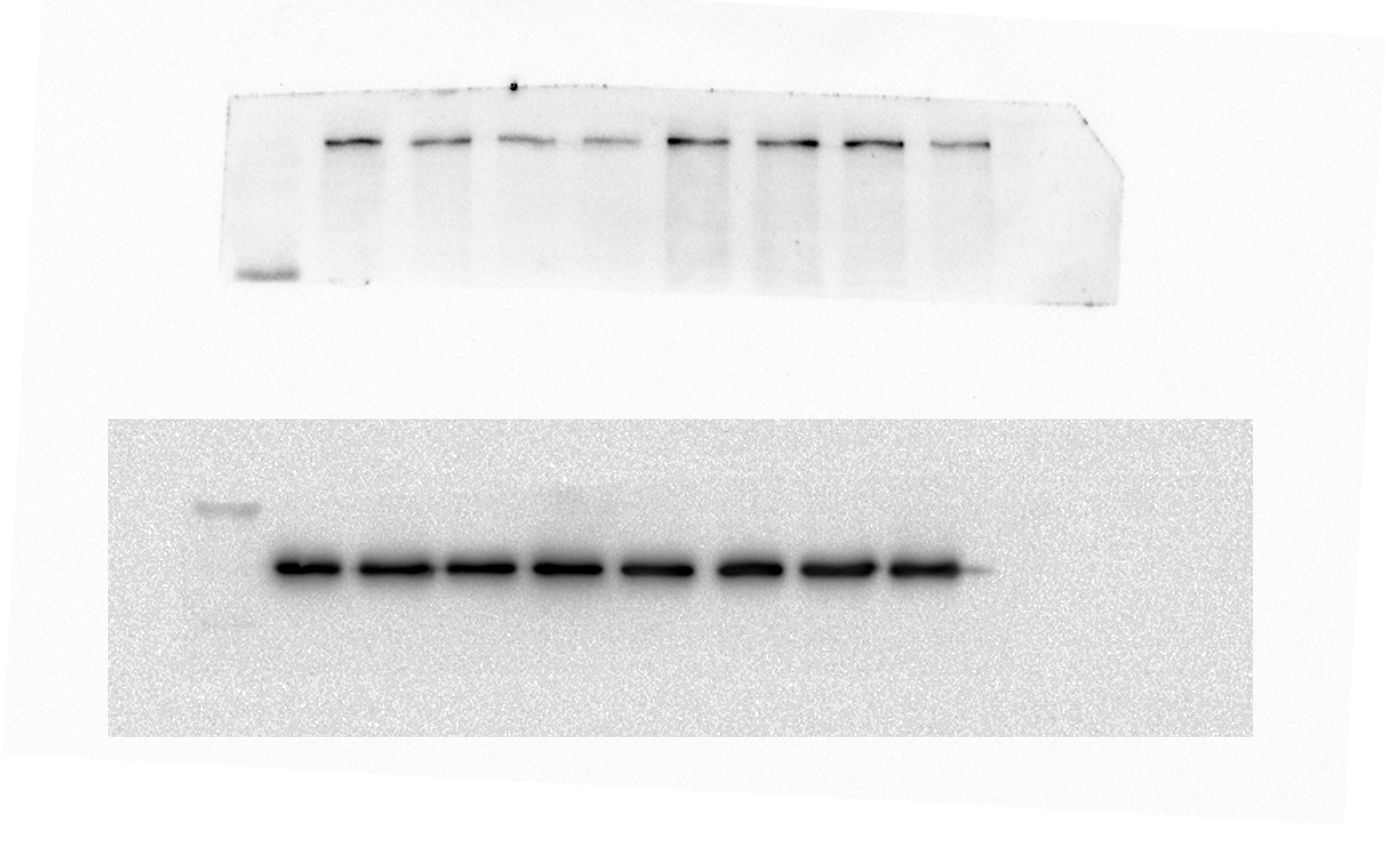

Supplement: Supplemental Information 5 [file peerj-10-13402-s005.zip › WB raw data/fig3/fig 3D 8-9.tif]

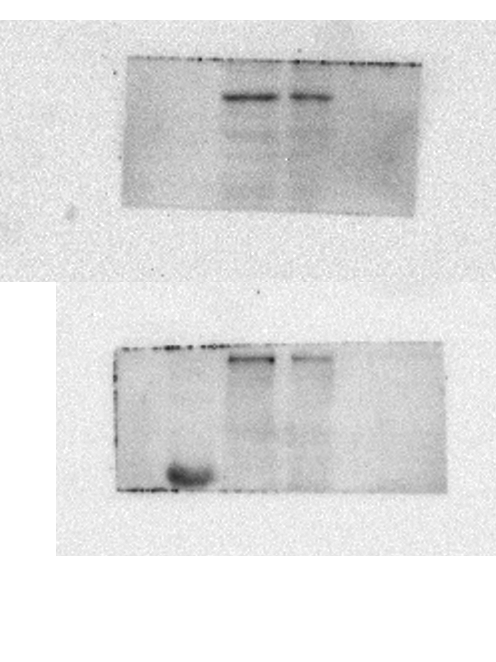

Supplement: Supplemental Information 5 [file peerj-10-13402-s005.zip › WB raw data/fig3/fig3 C 6-7.tif]

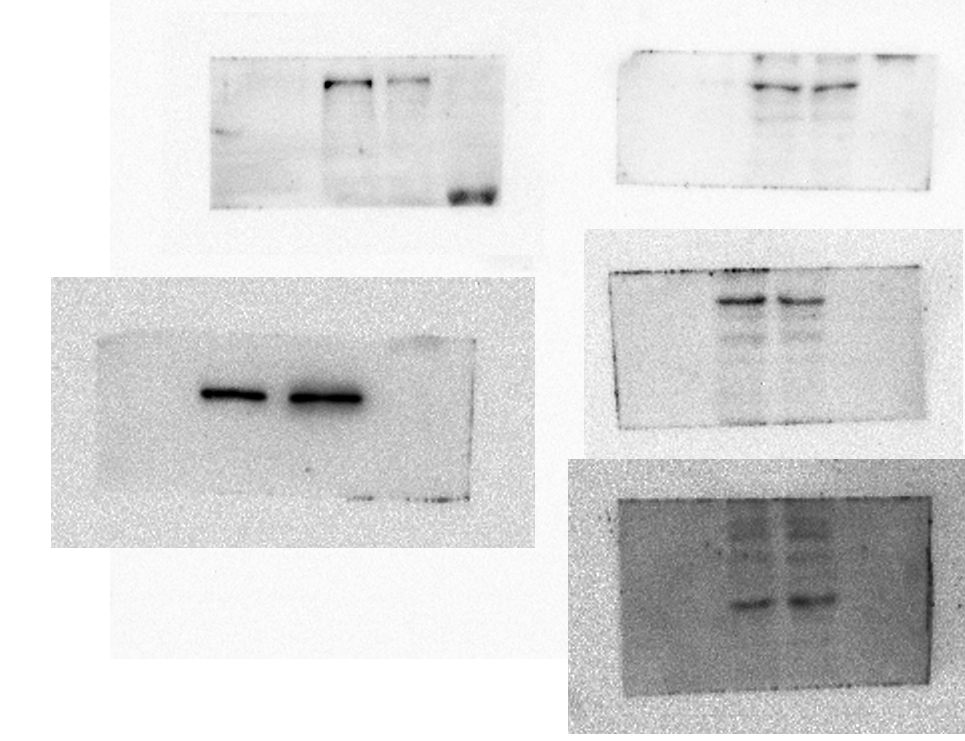

Supplement: Supplemental Information 5 [file peerj-10-13402-s005.zip › WB raw data/fig3/fig3A 1-5.tif]

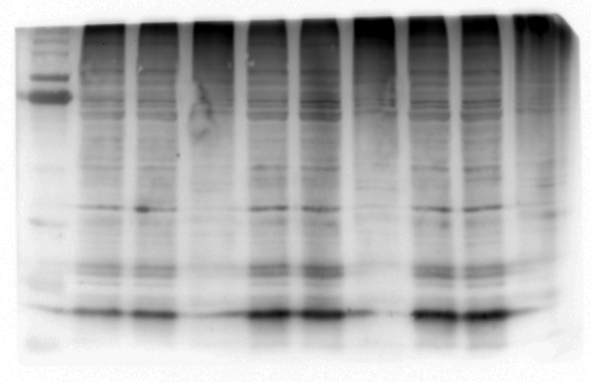

Supplement: Supplemental Information 5 [file peerj-10-13402-s005.zip › WB raw data/fig3/fig3f 10 lane6-7.png]

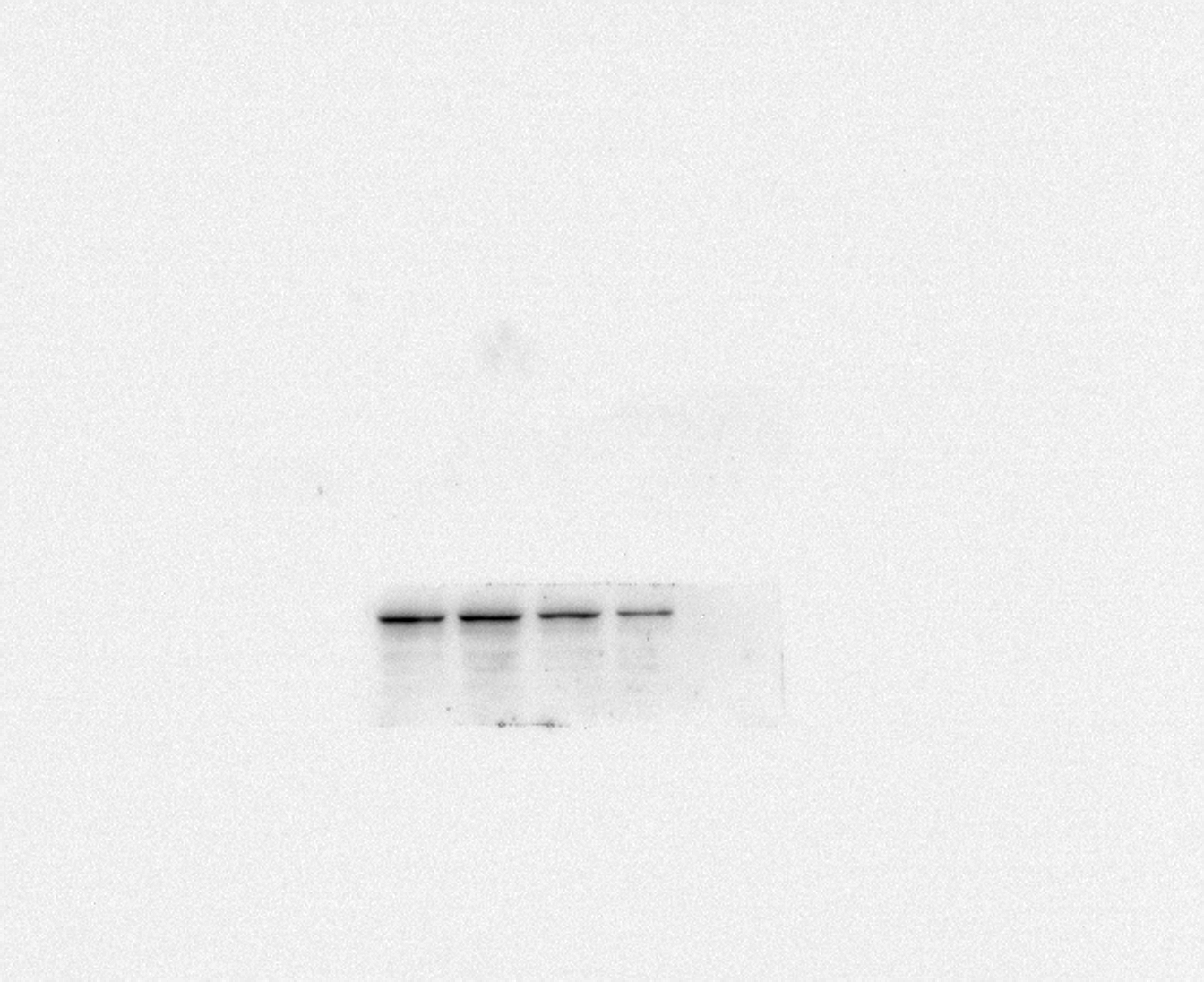

Supplement: Supplemental Information 5 [file peerj-10-13402-s005.zip › WB raw data/fig3/fig3f lane1-2 11.Tif]

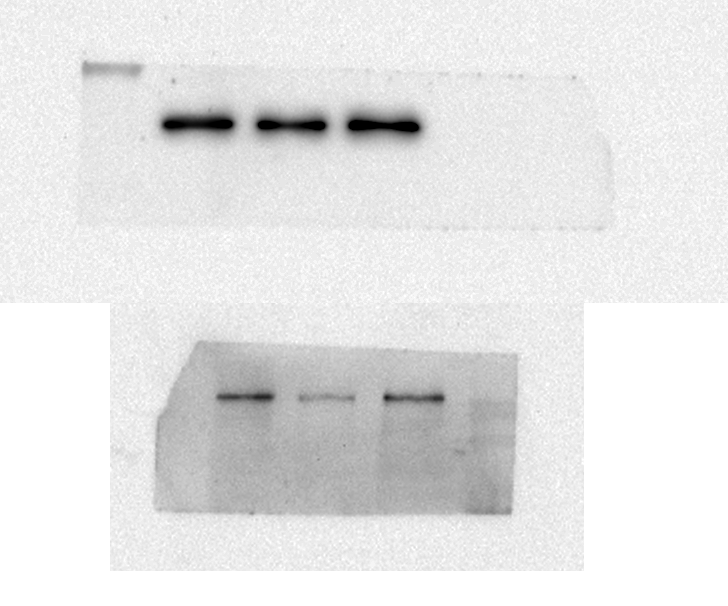

Supplement: Supplemental Information 5 [file peerj-10-13402-s005.zip › WB raw data/fig3/fig3G 12-13.tif]

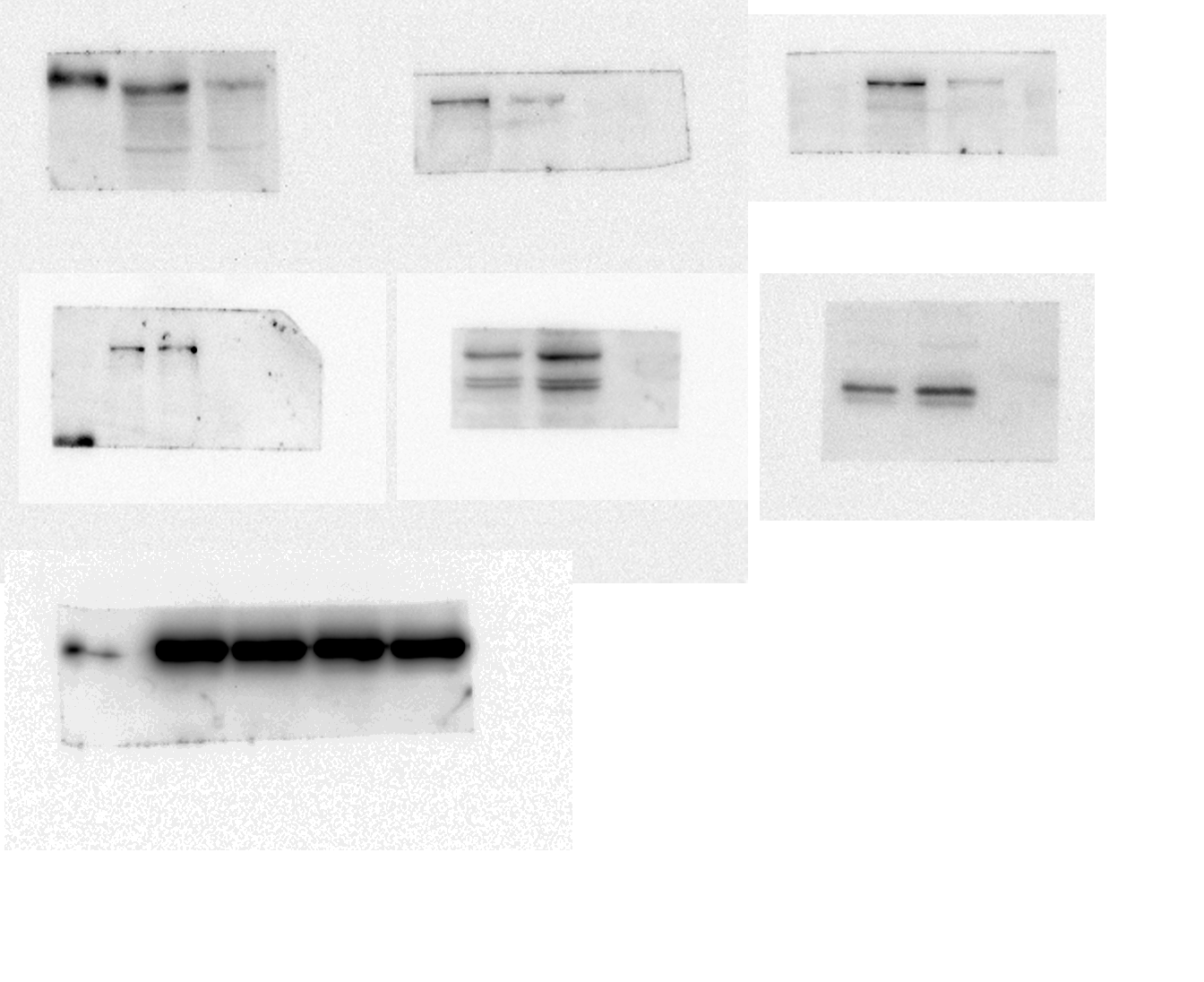

Supplement: Supplemental Information 5 [file peerj-10-13402-s005.zip › WB raw data/fig4/fig4 b+d 1-7.tif]

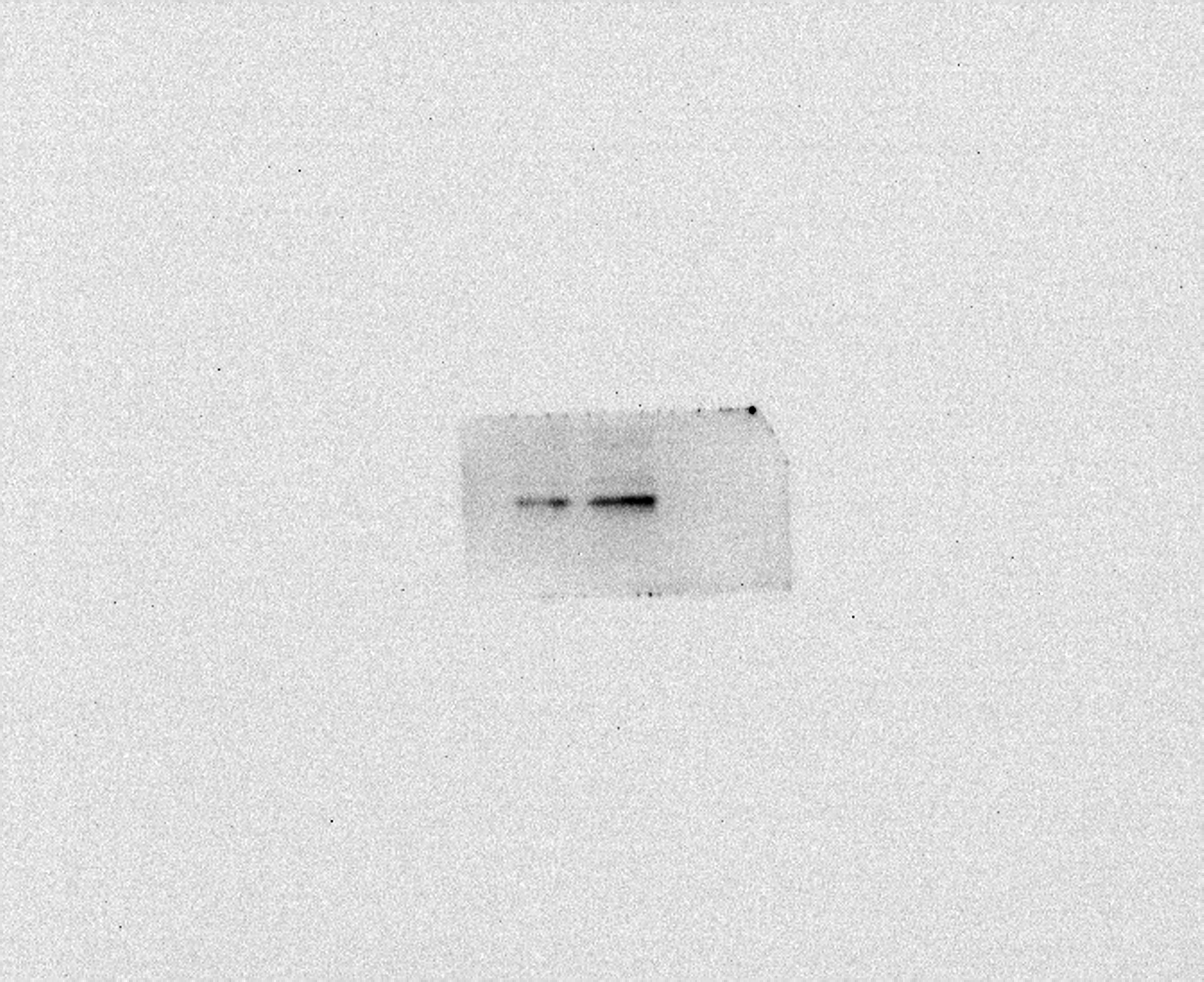

Supplement: Supplemental Information 5 [file peerj-10-13402-s005.zip › WB raw data/Supporting figuresS1/Supporting figS1-1.Tif]

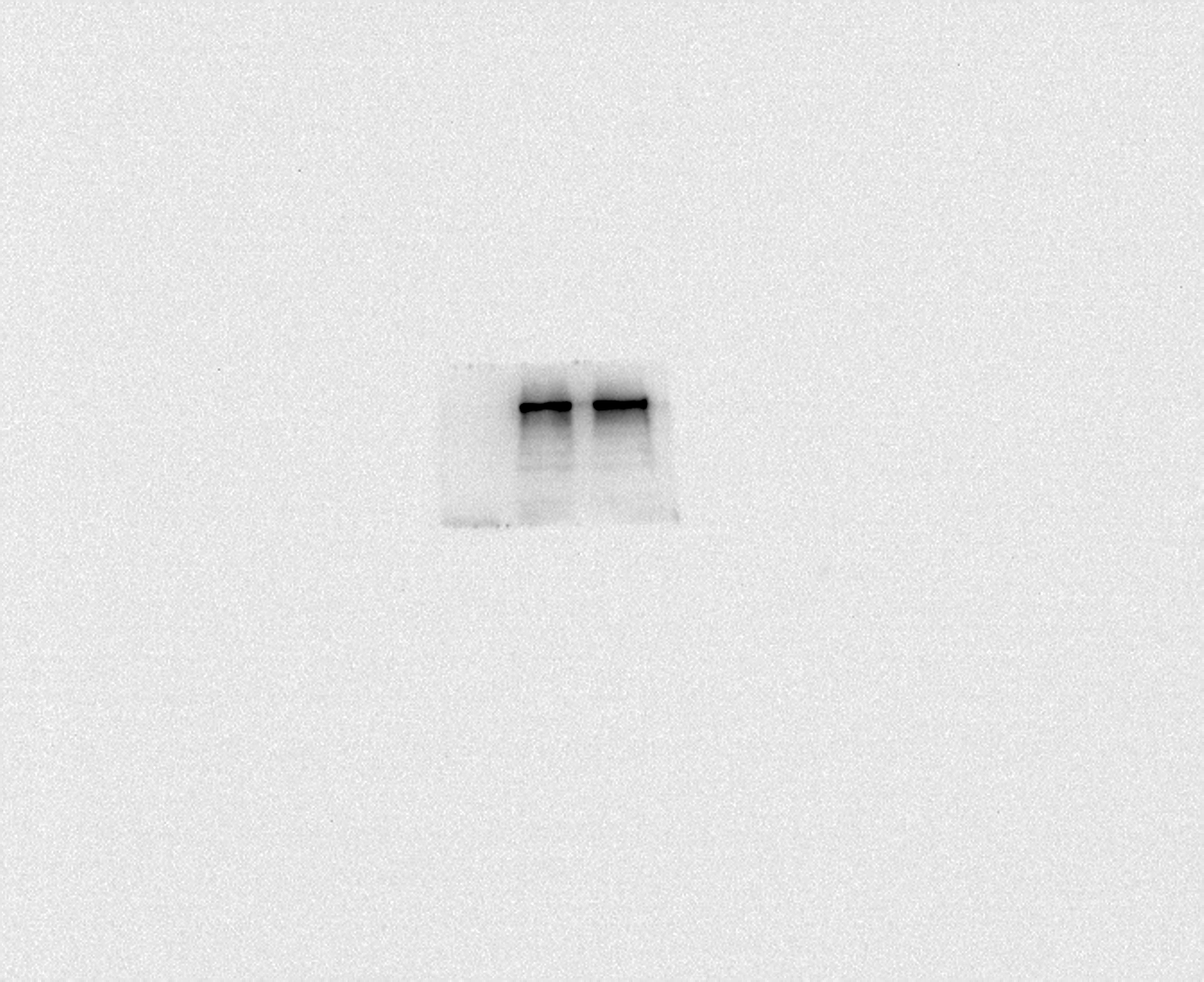

Supplement: Supplemental Information 5 [file peerj-10-13402-s005.zip › WB raw data/Supporting figuresS1/Supporting figS1-2.Tif]

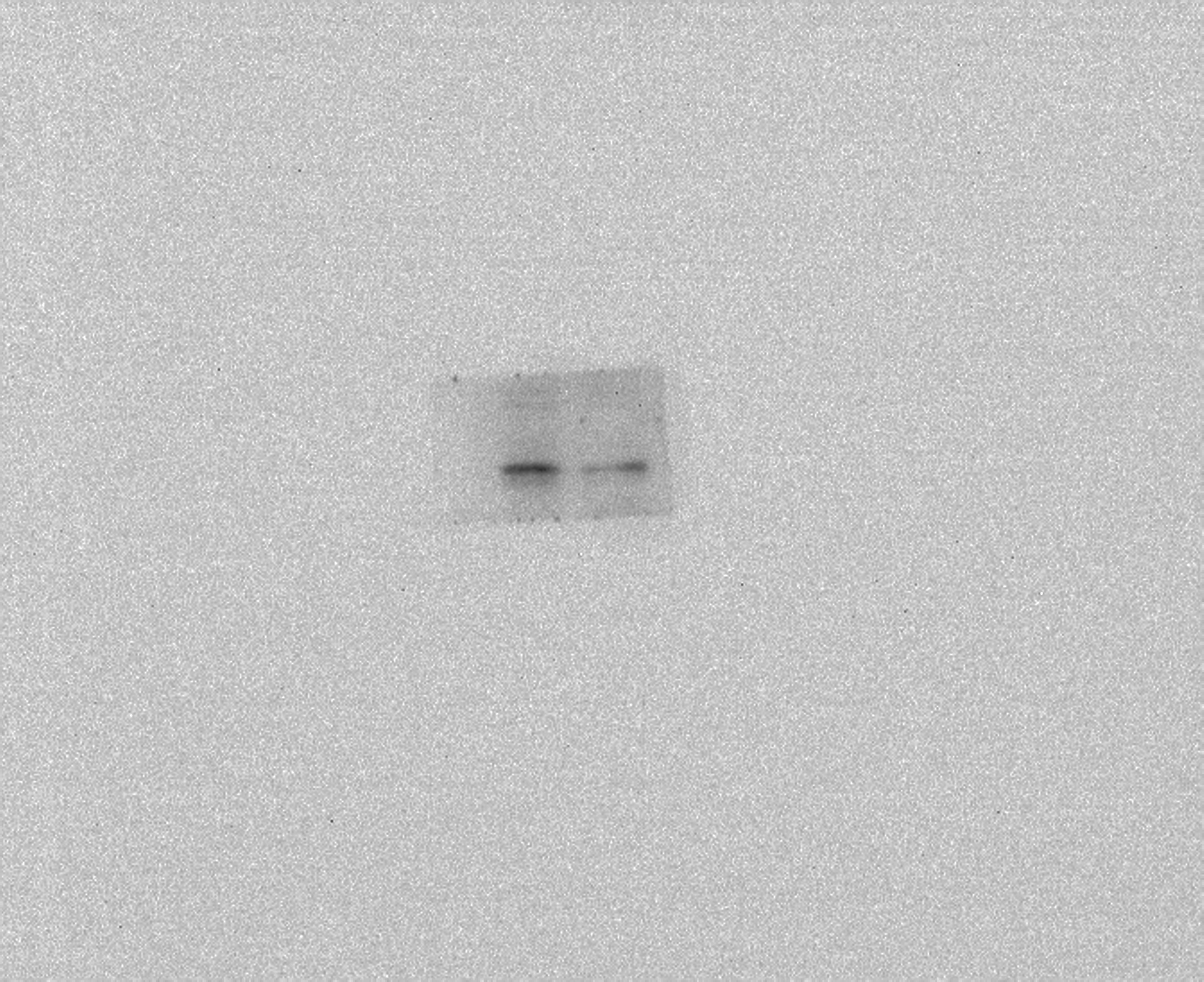

Supplement: Supplemental Information 5 [file peerj-10-13402-s005.zip › WB raw data/Supporting figuresS1/Supporting figS1-3.Tif]

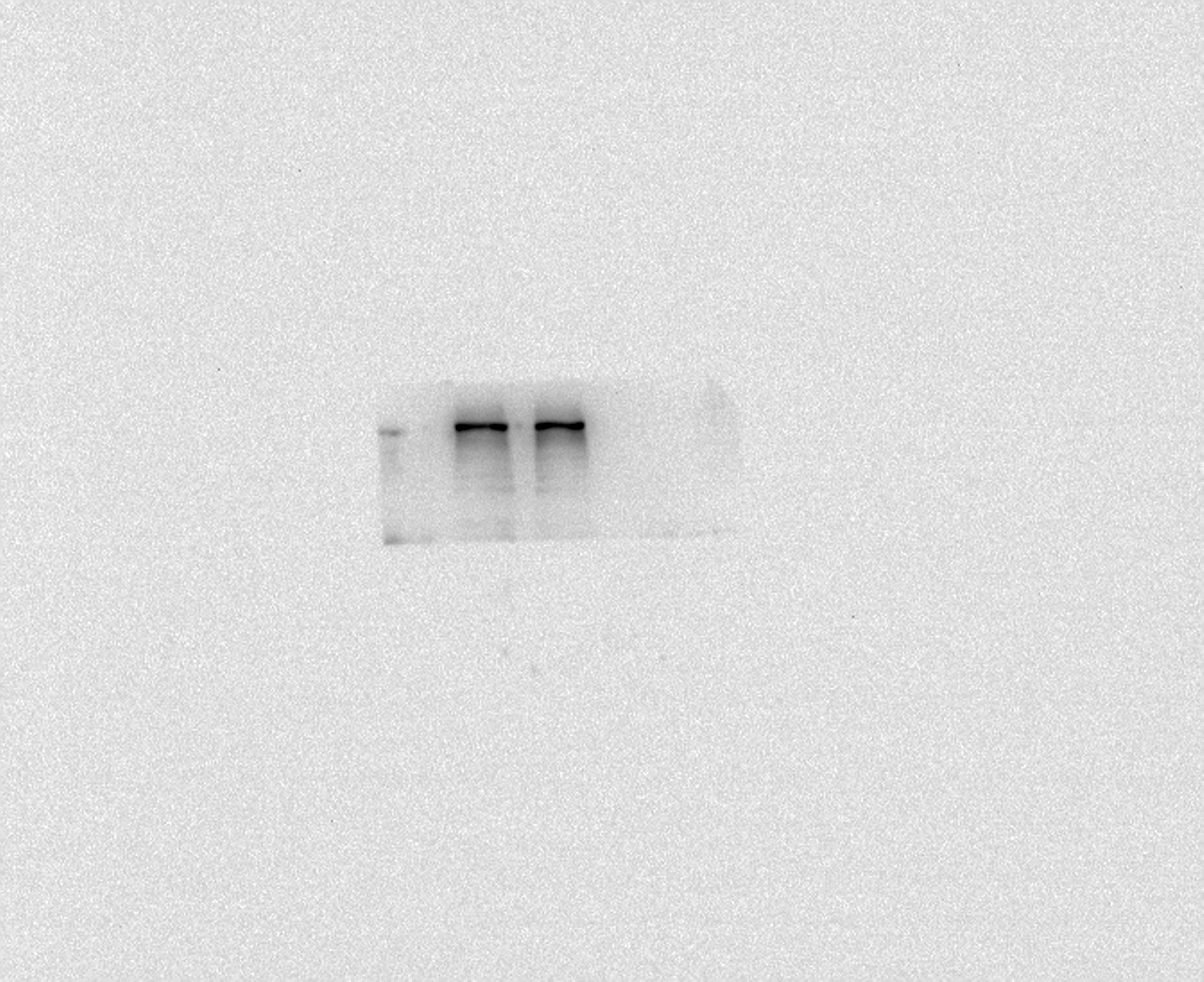

Supplement: Supplemental Information 5 [file peerj-10-13402-s005.zip › WB raw data/Supporting figuresS1/Supporting figS1-4.Tif]
